# Sources of Heterogeneity in Functional Connectivity During English Word Processing in Bilingual and Monolingual Children

**DOI:** 10.1162/nol_a_00092

**Published:** 2023-04-11

**Authors:** Xin Sun, Rebecca A. Marks, Rachel L. Eggleston, Kehui Zhang, Chi-Lin Yu, Nia Nickerson, Valeria Caruso, Tai-Li Chou, Xiao-Su Hu, Twila Tardif, James R. Booth, Adriene M. Beltz, Ioulia Kovelman

**Affiliations:** Department of Psychology, University of Michigan, Ann Arbor, MI, USA; Department of Psychology, University of British Columbia, Vancouver, Canada; Department of Brain and Cognitive Sciences, Massachusetts Institute of Technology, Cambridge, MA, USA; Department of Psychology, National Taiwan University, Taipei, Taiwan; Department of Psychology and Human Development, Vanderbilt University, Nashville, TN, USA

**Keywords:** brain development, functional connectivity, bilingualism, children, fNIRS, individual differences

## Abstract

Diversity and variation in language experiences, such as bilingualism, contribute to heterogeneity in children’s neural organization for language and brain development. To uncover sources of such heterogeneity in children’s neural language networks, the present study examined the effects of bilingual proficiency on children’s neural organization for language function. To do so, we took an innovative person-specific analytical approach to investigate young Chinese-English and Spanish-English bilingual learners of structurally distinct languages. Bilingual and English monolingual children (*N* = 152, *M*(*SD*)_age_ = 7.71(1.32)) completed an English word recognition task during functional near-infrared spectroscopy neuroimaging, along with language and literacy tasks in each of their languages. Two key findings emerged. First, bilinguals’ heritage language proficiency (Chinese or Spanish) made a unique contribution to children’s language network density. Second, the findings reveal *common* and *unique* patterns in children’s patterns of task-related functional connectivity. Common across all participants were short-distance neural connections within left hemisphere regions associated with semantic processes (within middle temporal and frontal regions). Unique to more proficient language users were additional long-distance connections between frontal, temporal, and bilateral regions within the broader language network. The study informs neurodevelopmental theories of language by revealing the effects of heterogeneity in language proficiency and experiences on the structure and quality of emerging language neural networks in linguistically diverse learners.

## INTRODUCTION

Early language experiences shape a child’s mind and brain while also laying foundations for reading (Werker & Hensch, 2015). Bilingualism offers enriched linguistic experiences that add to the heterogeneity in children’s neural organization for language and reading acquisition (Hernandez et al., 2019). To better capture the developing neural heterogeneity for language processing, the present study utilized a person-specific network mapping approach to characterize sources of heterogeneity in children’s emerging neural pathways for English and to identify how these language neural networks are influenced by early bilingual experiences with Spanish or Chinese.

According to the Neuroemergentist Framework, complex neurocognitive processes develop out of interactions between an individual’s *expertise* and the environmental *ecosystem* (Claussenius-Kalman et al., 2021). Bilingual development thus stems from dynamic communications between individuals’ developing cognitive skills (e.g., attention and working memory) as well as their bilingual experiences and contexts (e.g., linguistic and orthographic features and contexts of use). As a result, bilinguals may form different patterns of neural organization for language processing in relation to monolinguals and/or bilinguals of different language groups. For instance, a meta-analysis found that bilinguals speaking a language with more predictable sound-to-print mapping (e.g., French, as compared to English) rely on enhanced phonological networks (i.e., bilateral temporal regions). In contrast, those speaking a language with less predictable associations between language sounds and printed form (e.g., Chinese, as compared to English) rely on enhanced networks for lexical integrations (i.e., left middle/inferior frontal regions; Liu & Cao, 2016). Moreover, connectivity studies have found that, due to the increased cognitive demands of bilingual coordination, compared with monolinguals, bilinguals form enhanced connectivity between bilateral inferior frontal regions, as well as between the basal ganglia and the frontal cortex, which guides language perception, comprehension, and cognitive control (Berken et al., 2016; Marian et al., 2017).

Most prior research has approached bilingual brain development with group averages. However, variations in bilingual experiences may yield meaningful variability in the neural networks within groups. To advance the understanding of *heterogeneity* in neural mechanisms of spoken language processing, and how they are influenced by bilingualism, we examined the functional connectivity of cortical networks for spoken word recognition. Prior work has shown that children’s language and reading proficiency are positively associated with strengthened neural connectivity along key neural pathways of language processing (Skeide et al., 2016; Yeatman et al., 2011). Moreover, these neural networks develop as a function of language experience, including bilingualism (Ip et al., 2017; Kovelman et al., 2008; Marian et al., 2017). In other words, bilingual experiences contribute to the neural network heterogeneity of language development (Claussenius-Kalman et al., 2021; Hernandez et al., 2019). Using a person-specific approach, we aimed to uncover sources of individual variation in the development of neural networks that support language and literacy development.

### The Developing Neural Basis for Spoken Word Processing

Spoken words are comprised of sound (phonological units) and meaning (semantic units). Proficient adult speakers typically engage two parallel processing streams that allow them to simultaneously consider the multifaceted nature of phonological and lexico-semantic representations during word recognition. In the adult brain, these are commonly represented as dorsal and ventral neural streams (Hickok, 2022; Hickok & Poeppel, 2007). The dorsal or phonological stream includes the dorsal aspect of the left inferior frontal gyrus (IFG) and superior temporal gyrus (STG), as well as the arcuate fasciculus (AF) fiber tract that connects those regions. The ventral or semantic stream includes the ventral aspect of the left IFG, the middle temporal gyrus (MTG), and the inferior fronto-occipital fasciculus that connects them (Su et al., 2018). These two parallel processing streams improve in their functionality over the course of children’s language development, as children learn to efficiently access both lexical and sub-lexical information. Children’s spoken language skills are linked to functional and anatomical strengths within and between these networks (Cao et al., 2008; Friederici et al., 2011; Skeide et al., 2016; Yeatman et al., 2011; Yu et al., 2018).

Functional connectivity studies reveal how brain regions work together during a language task and how these brain connections relate to developmental outcomes in language proficiency (Friederici et al., 2011; Jasińska et al., 2020; Qi et al., 2021; Xiao et al., 2016; Yu et al., 2018, 2021). This research generally suggests a gradual shift in the development of inter- (between) and then intra- (within) hemisphere associations. For instance, Friederici et al. (2011) examined functional connectivity in 6-year-old children and adults who were performing an auditory sentence comprehension task during functional magnetic resonance imaging (fMRI). Findings revealed that younger children formed stronger functional connections between the left frontal regions and their right hemisphere homologs than adults. In contrast, adults showed stronger connectivity between the left frontotemporal regions. This and similar findings (Enge et al., 2020; Weiss-Croft & Baldeweg, 2015) exemplify the merits of functional connectivity research in revealing changes in language development, paving the way for more nuanced inquiries into sources of heterogeneity of such change.

### Connecting Spoken Language Networks to Reading

Learning to read requires children to connect their understanding of spoken words to orthography, or written symbols. Therefore, neural networks for spoken language are essential for children’s behavioral outcomes such as emergent literacy (Jasińska et al., 2020; Yu et al., 2018, 2021). For instance, Jasińska et al. (2020) examined the longitudinal effects of functional connectivity in 4-year-old children who passively listened to words during functional near-infrared spectroscopy (fNIRS). Findings revealed that children who exhibited stronger functional connectivity during this auditory task between the left IFG and right STG regions at age 4 years had better reading proficiency a year later. Building upon this and similar prior findings (Qi et al., 2021), we aimed to advance beyond the traditional functional correlation methods that average across diverse speakers. Here we estimate individualized neural networks to better capture sources of heterogeneity in children’s emerging neural architecture for language and how the neural networks speak to children’s developing language and literacy skills (Arredondo et al., 2022; Beltz et al., 2016).

### Individual Differences in the Neural Connectivity for Language in Bilingual Children

Neuroimaging research on bilingualism often finds connectivity differences between bilingual and monolingual populations. These examinations include both anatomical connectivity as studied through white matter tracts (García-Pentón et al., 2014; Mohades et al., 2012) and resting-state functional connectivity (Berken et al., 2016; Sun et al., 2019; Thieba et al., 2019). For example, in an anatomical diffusion tensor imaging study, Gao et al. (2022) examined the relation between bilingual proficiency and white matter tracts in Chinese-English bilingual children raised in China. Findings revealed that children with thicker AF tracts around left STG regions had better word reading proficiency in both English and Chinese. The AF is a tract that connects frontal and temporal language regions and generally increases in its thickness over the course of language development (Skeide & Friederici, 2016). Such neuroanatomical findings support the idea that there is a relation between bilingualism factors and neural connections critical for language processing (Bialystok et al., 2012).

Resting-state connectivity studies ask participants to stay awake while they are not engaged in any given task to reveal a presumed default state of brain operations. A resting-state fMRI study found that adults with early bilingual exposure (before age 5) showed stronger intrinsic functional connectivity between the left and right IFG regions and between left IFG and prefrontal regions than later-exposed bilinguals (Berken et al., 2016). The findings suggest that early bilingual exposure influences the neural organization of the frontal lobe network essential for language control (Berken et al., 2016; Bialystok et al., 2012). The advantage of resting-state neuroimaging studies is that they can capture spontaneous signals that do not tie to a specific mental state (i.e., a task). Nevertheless, non-task resting-state paradigms may lack empirical benefits such as sensitivity to brain-behavior associations (Finn, 2021). To the best of our knowledge, no prior study has examined bilingual children’s functional connectivity networks while participants engage in a language task, which is a knowledge gap we aim to fill in the present work.

Another important but understudied issue is how to best depict the neural networks of language processing for bilingual children. Much current knowledge about bilingualism stems from analytical approaches that have dichotomized bilinguals versus monolinguals or otherwise categorized different groups of bilinguals, such as splitting by age of exposure or proficiency (e.g., Liu & Cao, 2016; Sulpizio et al., 2020). However, bilinguals can differ in many ways. Newly emerging research thus advocates for approaches that leverage the heterogeneity of bilingual profiles to better understand bilingualism (Luk & Bialystok, 2013; Marian & Hayakawa, 2021). The present work thus adopts a person-specific approach to examine such heterogeneity of functional connectivity for language in relation to children’s bilingual language and reading development.

### Examining Person-Specific Neural Network With GIMME

Person-specific analytical approaches, such as group iterative multiple model estimation (GIMME; Gates & Molenaar, 2012), advance upon conventional data analysis methods that average across heterogenous individuals by instead identifying connections among a priori regions of interest (ROIs) that are shared across participants (group level), across a subgroup of participants (subgroup level), as well as connections that are unique to one or some individuals (individual level). In this way, GIMME networks capture both the broad homogeneity of the group and the heterogeneity of individuals. Specifically, GIMME uses a data-driven approach to yield person-specific directed connectivity maps; GIMME begins with a null network and then adds connections among ROIs that are meaningful (i.e., significant) for at least 75% of participants to all participants’ networks followed by adding connections that are meaningful for a subgroup of participants. Finally, GIMME adds connections that are meaningful just to an individual. Connections are added until each person’s network represents their observed data well and has person-specific weights. Simulation studies suggest that GIMME shows exceptional robustness in modeling heterogeneous data compared to nearly 40 alternative methods, and it has been applied to a wide range of psychological studies with neuroimaging data (Dotterer et al., 2020; Gates & Molenaar, 2012; Goetschius et al., 2020; Lane et al., 2019; Price et al., 2020). Altogether, as a data-driven network mapping approach, GIMME addresses the limitations of traditional group-oriented approaches that rely on averages while also allowing for both group-level inferences and accurate reflections of individual-level heterogeneity.

GIMME has been used to examine the functional connectivity of the attention networks in bilingual children. Arredondo et al. (2022) used GIMME to estimate bilingual and monolingual children’s neural connectivity with the attention network task among six pre-specified left superior, middle frontal, as well as parietal brain channels with fNIRS. GIMME identified two subgroups, one that consisted of almost all monolinguals (92%) and half of the bilinguals (54%), and another that consisted of a small portion of monolinguals (8%) and the other half of bilinguals (46%). Notably, the bilinguals in the first group were more English-dominant (i.e., “monolingual-like”), whereas the bilinguals in the second group had more balanced proficiency across their two languages. Importantly, the second group also had significantly higher network density (i.e., number of connections) centered around the left frontal regions compared to the first group, which also corresponded to higher attention task accuracy. These results suggest more complex attentional neural networks for early bilingual children with more balanced dual language proficiency. In sum, GIMME has been shown to be an effective approach for understanding sources of heterogeneity in the neural organization of cognitive functions in bilingual children, but many questions remain unanswered, particularly regarding neural networks during a language task.

### The Present Study

The current study employed GIMME analysis of fNIRS data to examine the effects of early and systematic bilingual experiences on children’s emerging neural architecture for language processes and their relation to literacy development. The participant groups included children (ages 5–10 years) who were English monolinguals, Chinese-English bilinguals, or Spanish-English bilinguals, all experiencing English-dominant education in the US. The bilinguals were exposed to a heritage language (Chinese or Spanish) at home from birth, to English around age 2, and were capable of reading words/characters in their heritage languages. The study specifically asked participants to complete an auditory word-processing task during fNIRS neuroimaging. Children heard three words and were asked to identify the two words that shared a unit of meaning (morpheme) while ignoring a phonological distractor (e.g., *bedroom*, *classroom*, mushroom). The task probed children’s ability to analyze words’ lexico-semantic and phonological constituents necessary for successful word processing. The ability to operate upon words’ sound and meaning units is thought to support children’s emergent literacy (Kuo & Anderson, 2006; Sun, Zhang, Marks, Nickerson, et al., 2022).

Functional connectivity analyses were performed with a priori brain regions of language processing, including bilateral frontal and left temporal areas. These regions have been identified as essential to spoken word recognition by previous research (Enge et al., 2020; Friederici et al., 2011; Jasińska et al., 2020) as well as for the current sample (see Sun et al., 2023, for the functional activation patterns). We used GIMME to ask two experimental questions. First, we asked: What is the relation between individual differences in functional connectivity for word processing and children’s literacy skills in English? To answer this question, we applied GIMME to identify potentially different groups of learners. We then examined the relationship between children’s English proficiency and their network characteristics, focusing on network density within the identified language regions, which is thought to reflect the quality of the language network (Jasińska et al., 2020). Second, we asked: How do bilinguals’ heritage language skills contribute to the neural network quality of English word processing? We predicted significant associations between children’s neural networks and behavioral profiles, and therefore examined the brain-behavioral associations between children’s connectivity network patterns and their proficiency in English and their heritage language. Together, the goal of the study was to inform our understanding of the effects of bilingualism and sources of heterogeneity in children’s emergent language networks.

## MATERIALS AND METHODS

### Participants

Participants were 152 children (75 girls, *M*_age_ = 7.71 years, *SD*_age_ = 1.32, age range = 5.12–10.19) recruited from southeast Michigan, USA. Participants were all typically developing without a history of developmental delays in language or literacy, deficits in hearing, or other neurological or physical disorders. All children grew up in the United States, attended English-only schools, and were proficient English users, as determined by standard vocabulary scores over 85 on the Peabody Picture Vocabulary Test 5 (PPVT-5; Dunn, 2019). All three groups were matched on age, gender, grade distribution, maternal education, and non-verbal working memory (see Table 1). Parents and children provided appropriate informed consent or assent and received $40 for their participation. The study was approved by the Institutional Review Board for research with human subjects.

**Table 1.** Demographics and English task performance by participant group

		English monolingual *N* = 54	Spanish bilingual *N* = 50	Chinese bilingual *N* = 48	*p*
		*M*(*SD*) or *n*	*M*(*SD*) or *n*	*M*(*SD*) or *n*	
*Demographics*					
Age		7.66 (1.32)	7.84 (1.22)	7.63 (1.44)	0.921
Grade^a^	K	14	6	16	0.051
	1	9	18	9	
	2	16	7	7	
	3	8	12	11	
	4	7	5	7	
Working Memory^b^		7.15 (2.43)	7.36 (2.15)	7.76 (2.64)	0.443
Maternal Education^c^		88.9%	84.0%	95.8%	–

*English task performance*					
Vocabulary		158.40 (26.84)	144.30 (28.14)	145.09 (32.95)	0.022
Phonological awareness		21.42 (7.79)	23.62 (7.23)	22.63 (7.48)	0.401
Morphological awareness		25.31 (11.32)	24.42 (10.01)	24.54 (11.18)	0.712
Word reading		46.75 (16.79)	48.54 (15.27)	50.47 (14.36)	0.234
Reading comprehension		26.12 (9.18)	24.64 (6.88)	27.22 (8.35)	0.540
Sentence reading fluency		37.55 (17.68)	34.78 (18.49)	40.71 (21.39)	0.335

*fNIRS task accuracy (%)*		79.6 (9.3)	77.2 (10.2)	81.1 (9.8)	0.336

^a^ Grade distribution used a *χ*^2^ test with *df* = 8.

^b^ Measured by a backward digit span task (Wechsler, 2014).

^c^ % bachelor’s degree or above.

Participants had diverse language experiences: 35.5% were English monolinguals (*N* = 54), while the remaining 64.5% were either bilingual English-Chinese (*N* = 48) or English-Spanish (*N* = 50) speakers. According to the parental reports, the bilinguals were exposed to their heritage language (Chinese or Spanish) from birth at home and with at least one parent considering themselves to be a native speaker of the language. Bilingual children were also systematically exposed to English before or beginning at age two (i.e., used English regularly in contexts such as daycare or preschool). Heritage language vocabulary was used to identify children’s heritage language proficiency. Of note is that although they provide some information about language proficiency, both standard scores of Chinese and Spanish should be interpreted cautiously, as the norm of the Chinese vocabulary task was based on children growing up in Taiwan in 1988 (PPVT–Revised; Dunn & Dunn, 1998), and the Spanish norm was based on children growing up in Mexico and Puerto Rico in 1986 (Test de Vocabulario en Imágenes Peabody [TVIP]; Dunn et al., 1986). To account for the limitation from the norm and to capture variations in the bilingual heritage speakers, no participants were excluded on account of low heritage language vocabulary. Nonetheless, all Spanish bilingual participants had a Spanish receptive vocabulary standard score above 70, and 93% of Chinese bilingual participants passed this threshold in Chinese receptive vocabulary.

### Measures and Procedure

All participants completed the full battery of behavioral and neuroimaging tests during a single laboratory visit. Participants completed language and literacy measurements in each of their languages including vocabulary, phonological awareness, word reading, reading comprehension and fluency, and morphological awareness. Across languages, these tasks were maximally matched by either using similar standardized assessments that are already available (e.g., vocabulary across languages) or building measures that were maximally similar across language assessments (e.g., an experimental elision task in Chinese to match the Spanish and English versions). In selecting language measures, we took into account the need to make these measures maximally comparable and the fact that the measures need to capture specific features of each language. We therefore acknowledge that the tests are maximally comparable in capturing respective skills, but not identical across languages. All self-developed tasks are openly available and can be found in Sun, Zhang, Marks, Karas, et al. (2022). For the current study, data and codes can be found at https://osf.io/uv3t6/?view_only=46569a15ebd241808a01d51f550c65dd.

#### Vocabulary

Vocabulary was tested with the Peabody Picture Vocabulary Test in English (PPVT-5; Dunn, 2019); in Chinese (PPVT-Revised; Dunn & Dunn, 1998); and in Spanish (TVIP; Dunn et al., 1986). Children saw four pictures, heard a word, and selected the picture that best describes the word.

#### Phonological awareness

Phonological awareness was measured with a sound elision task in which children heard a word and were asked to omit a phonetic unit from the word (e.g., “*Cat* without /k/ is ___.” [at]). The English task used the Elision subtest from the Comprehensive Test of Phonological Processing (Wagner et al., 1999), the Spanish task used the Test of Phonological Processing in Spanish (Francis et al., 2001), and the Chinese task was adapted from Newman et al.’s (2011) measure with the same paradigm.

#### Morphological awareness

For this task, we aimed to tap into lexical morphological awareness across languages and capture morphological features of each language (i.e., compound structures in Chinese and both compound and derivational structures in English/Spanish). In English, we used the Early Lexical Morphology Measure (Marks, Labotka, et al., 2022), which includes compound and derivational words. Children were asked to complete a sentence with part of a given word (e.g., “*Football*. Ouch! You stepped on my ____.” [foot]; “*Friendly*. She is my best ___.” [friend]). A parallel task was used in Spanish (Marks, Sun, et al., 2022). In Chinese, a morphological construction measure was used (modified from Song et al., 2015). Children were asked to create a new word with a given word, for example, “Apple trees grow apples. What trees might grow bread? [bread trees].”

#### Word/character reading

Word/Character reading was measured by presenting a list of words/characters and asking children to read them aloud. The English task was the Letter-Word Identification subtest from Woodcock-Johnson IV (Schrank et al., 2014); the Spanish task was the Word Identification subtest from Batería III Woodcock-Muñoz (Muñoz-Sandoval et al., 2005); and the Chinese task was a self-developed measure (Sun, Zhang, Marks, Karas, et al., 2022).

#### Sentence reading fluency

Sentence reading fluency was measured using a 3-min timed task in which children read short sentences and indicate whether each sentence is true or false (e.g., “The sky is blue” is “True”; “The milk is black” is “False”). English and Spanish tasks used the Sentence Reading Fluency subtest from the Woodcock-Johnson IV (Schrank et al., 2014) and Woodcock-Muñoz (Muñoz-Sandoval et al., 2005), respectively, and the Chinese task was a self-developed measure (Sun, Zhang, Marks, Karas, et al., 2022).

#### Passage reading comprehension

Passage reading comprehension was tested in English and Spanish. They both used the Passage Comprehension Woodcock-Johnson IV (Schrank et al., 2014) and Woodcock-Muñoz (Muñoz-Sandoval et al., 2005), respectively. Passage-level reading comprehension was not measured in Chinese because the Chinese-speaking children were generally not able to read and comprehend passage-long texts in Chinese.

Table 1 displays children’s English task performance by bilingual group, and the three groups were maximally matched in these tasks except for English vocabulary (Monolinguals > Bilinguals, and the two bilingual groups did not differ). Table 2 displays all children’s task performance on the behavioral tasks by language. Note that the current sample included early exposed, simultaneous dual-language learners with relatively balanced bilingual proficiency, and it is typical for these children to show positive associations between skills of their two languages (Chung et al., 2019; Wagley et al., 2022).

**Table 2.** Behavioral and neuroimaging task performance by language (*M*s and *SD*s)

	English task	Chinese task			Spanish task		
	Score	Score	*r*	Partial *r*^c^	Score	*r*	Partial *r*^c^
*Oral Language Measures*							
Vocabulary	149.6 (29.9)	54.8 (29.2)	0.15	0.03	67.7 (19.4)	0.70***	0.46***
Phonological awareness	22.6 (7.5)	22.0 (9.6)	0.84***	0.79***	13.6 (6.2)	0.80***	0.79***
Morphological awareness	24.2 (10.8)	13.5 (6.3)	0.52***	0.27****	27.9 (13.3)	0.66***	0.59***

*Literacy Measures*							
Single word reading	48.6 (15.6)	17.44 (13.8)	0.44***	0.14	42.8 (20.1)	0.67***	0.47***
Reading comprehension	26.0 (8.3)	/	/	/	19.9 (7.9)	0.69***	0.44***
Sentence reading fluency	37.2 (18.6)	11.93 (7.7)	0.54***	0.36***	26.1 (15.7)	0.76***	0.69***

*Note*. *r* = the English-Spanish or English-Chinese bivariate correlation of the respective language and literacy measure. ^c^ = partial correlation controlling for age. *p < 0.05, **p < 0.01, ***p < 0.001.

### Neuroimaging Word Processing Task

**Figure 1.**
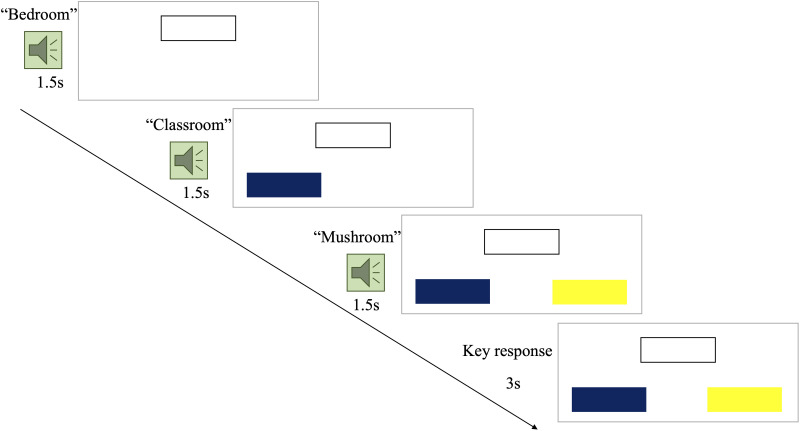
An example trial of the fNIRS word-processing task. For each trial, participants first hear the target word (e.g., “bedroom”) and see a white box on the top of the screen, then they hear two words of choice (e.g., “classroom,” “mushroom”) and simultaneously see a blue and a yellow box, respectively.

The neuroimaging word processing task assessed children’s morpho-semantic word knowledge using a lexical decision task. During each task item, children heard three words, one target word followed by two words of choice. Children were asked to select the word that shared either a root or derivational morpheme with the target word. Example items are *bedroom*, *classroom*, *mushroom* (shared root morpheme *-room*); *disagree*, *dishonest*, *distance* (shared derivational morpheme *dis-*). In the control condition, one of the choice words matched the target in its entirety (whole word match: *country*, *country*, *dentist*). The task followed a block design with 12 four-trial blocks (48 items in total). During each trial, participants heard three words and were instructed to select which of the last two matches the first with a keypress. Each trial took 7.5 s and the whole task took about 7.2 min. An example item is shown in Figure 1. All task items are available in Table S1 in the Supporting Information available at https://doi.org/10.1162.nol_a_00092.

### fNIRS Data Acquisition

**Figure 2.**
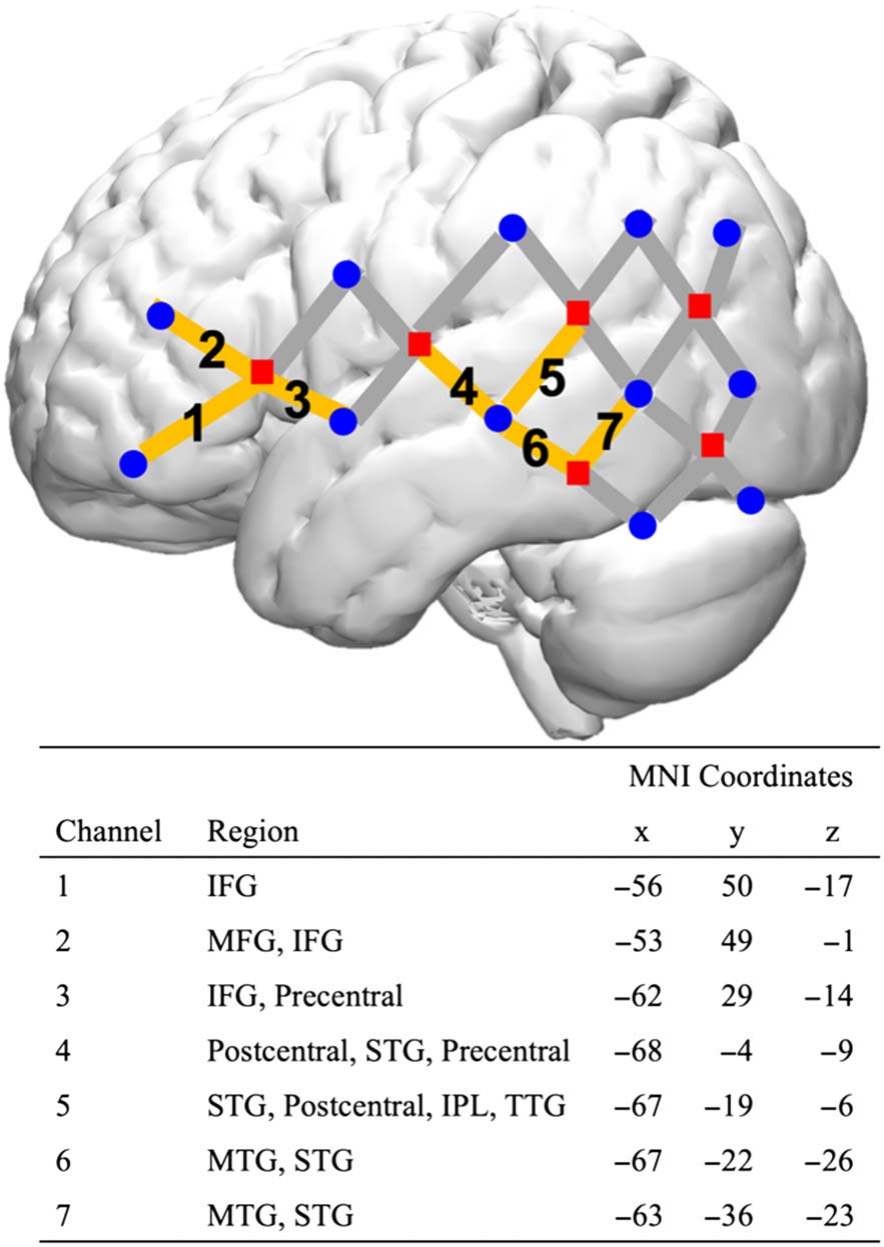
fNIRS probe setup and GIMME ROI location. Each ROI is formed by a source-detector pair. The 10 ROIs are bilateral C1, C2, C3, and left hemisphere C4, C5, C6, C7 (orange highlights). Red squares: light sources; blue circles: light detectors. IPL: inferior parietal lobe; TTG: transverse temporal gyri.

fNIRS data were collected using the TechEN-CW6 system (NIRSOptix, 2018) with 690 and 830 nm wavelengths and a 50 Hz sampling frequency. The fNIRS cap had 12 near-infrared light sources and 24 detectors that were symmetrically located on both hemispheres, yielding 46 source-detector data channels (23 per hemisphere; see Figure 2). The fNIRS channels aimed to capture key regions of language and reading networks, including frontal, temporal, and parietal regions. Of important note is that fNIRS is a surface-based neuroimaging method that may not provide the same level of precision as fMRI. Therefore, all references to anatomical locations are approximations of the neural regions maximally overlayed by specific channels. For the current investigation, brain region localizations captured by the fNIRS channels were co-registered using MRI as well as surface-based registration technologies. (For more information about the channel MNI localization, see Figure S1 in the Supporting Information and Hu et al., 2020). The depth of near-infrared light penetration was ∼3 cm, thus detecting cortical activities. fNIRS data for the current project are openly available on the Deep Blue Data repository and can be found in the data manuscript (Sun, Zhang, Marks, Karas, et al., 2022).

To ensure consistency in fNIRS cap placements across participants, trained experimenters follow standardized study protocols as established in fNIRS and electroencephalography fields to take head measurements and place caps. Specifically, experimenters first located participants’ nasion, inion, Fpz, and left and right pre-auricular points, and took the head circumferences. Next, F7, F8, T3, and T4 were anchored to their respective sources or detectors on the fNIRS cap. Experimenters then attached the fNIRS cap to participants’ scalps and inserted the optodes to their respective source or detector positions. Finally, experimenters conducted data quality control by checking the participant’s cardiac signal components and the signal-to-noise ratio among key channels of interest.

### Data Analysis

#### fNIRS data preprocessing

fNIRS data were analyzed with the NIRS brain AnalyzIR, a MATLAB-based toolbox (Santosa et al., 2018), as well as self-developed scripts. Data were first downsampled from 50 Hz to 2 Hz to fit the standard analysis protocols of GIMME (as recommended by Beltz & Gates, 2017, and done in Arredondo et al., 2022). Specifically, because GIMME conducts network mapping based on data temporal dynamics, data series with high frequency may exclusively yield high autoregressions, making it harder to detect connections between ROIs, which are often of primary interest (i.e., relationships between frontal and temporal regions; Beltz & Molenaar, 2015). Next, applying the modified Beer-Lambert Law, the optical density data was converted to hemoglobin concentration data. The data analysis focused on HbO signal as it contributes to about 76% of the fNIRS signal and the TechEN CW6 system obtains the HbO signal more reliably than HbR (Gagnon et al., 2012).

#### Regions of interest

We selected 10 ROIs with two steps. First, generally, ROIs should tap into key auditory word and morpho-semantic processes according to prior literature (e.g., Bulut, 2022; Enge et al., 2020; Ip et al., 2017). Thus, ROIs should include three main hubs, namely, frontal, superior temporal, and middle temporal regions. Second, specifically, ROIs should stay engaged when participants are working on the current task (for specific brain activation map, see Figure S2). The final ROIs included bilateral C1 (ventral IFG [vIFG]), bilateral C2 (middle frontal gyrus [MFG], and IFG), bilateral C3 (vIFG), left C4, and C5 (STG), and left C6 and C7 (MTG).

#### GIMME model fitting

GIMME builds person-specific connectivity networks with group-level, subgroup-level, and individual-level connections based on time-series data among a set of pre-determined ROIs (Lane et al., 2019). The connections can be contemporaneous, which depicts directed associations between ROIs at the same time points; and the connections can be lagged, which shows directed associations from a time point to its next time point within the same ROI or from one ROI to another (Beltz & Gates, 2017). For the current fNIRS data set, we focused on contemporaneous associations to better describe the cross-ROI relationships (for similar applications, see Goetschius et al., 2020). fNIRS data has high autocorrelations within a channel, which often yields lagged connections within each individual ROI and these connections typically do not provide much meaningful information but are important to model statistically (Smith et al., 2011, 2012).

The fNIRS HbO time-series data for each participant were extracted and submitted to the GIMME algorithm in R (Lane et al., 2017; https://cran.r-project.org/web/packages/gimme). GIMME first estimates a null model and gradually adds group-level connections that would significantly improve the model fit for 75% of the sample, according to Lagrange multiplier tests (criterion supported by simulations in Gates & Molenaar, 2012; Lane et al., 2019). After all group-level connections are added, GIMME then prunes connections that may no longer meet the 75% criterion. Next, GIMME ide”tifi’s subgroups using the Walktrap community detection algorithm and adds subgroup-level connections using a 50% criterion so that identification of a subgroup connection means significantly improving model fit for 50% of the subgroup, according to Lagrange multiplier tests (criterion supported by simulations in Lane et al., 2019). The last stage adds significant individual-level connections for a participant, according to Lagrange multiplier tests, until the network fits well. According to Brown (2014), models with excellent fit should have at least two out of four fit indices meet the following criteria: standardized root mean residual (SRMR) ≤ 0.05, comparative fit index (CFI) ≥ 0.95, root mean squared error of approximation (RMSEA) ≤ 0.05, and non-normed fit index (NNFI) ≥ 0.95.

#### Group and subgroup neural connectivity

Group-level connections and subgroup-level connectivity patterns were described and compared by the location of the connections and connection density by subgroup. To examine how subgroups differ from each other, we further compared participants’ in-scanner task accuracy as well as their language and literacy task performance across subgroups with one-way analysis of variance (ANOVA).

#### Person-specific neural network density

For each participant, network density was calculated by the number of connections within their neural network (Dotterer et al., 2020; Goetschius et al., 2020). To investigate how participants’ English language and literacy proficiency is associated with their brain networks, we ran bivariate and partial correlation analyses correlating network density with task performance, including neuroimaging task accuracy and individual standardized assessments of English (i.e., vocabulary, word reading, reading comprehension, sentence reading fluency, respectively), partial correlations controlling for age. To investigate how bilingual children’s heritage language proficiency is associated with their brain networks, for each bilingual group, we further conducted separate multiple regression analyses using heritage language vocabulary and word/character reading to predict children’s brain network density, controlling for age and English proficiency. We chose these two measures as indicators of heritage oral and reading proficiency, respectively. We excluded analyses with the sentence-level fluency reading task because many children were not able to read and comprehend full sentences in their heritage language (*N* = 15 Spanish and *N* = 22 Chinese children were not able to complete the task).

## RESULTS

The current GIMME analysis yielded well-fitting models across participants, with an average SRMR at 0.027, CFI at 0.962, RMSEA at 0.103, and NNFI at 0.940. We next report group-, subgroup-, and person-specific results in greater detail.

### Group-Level Neural Connections

**Figure 3.**
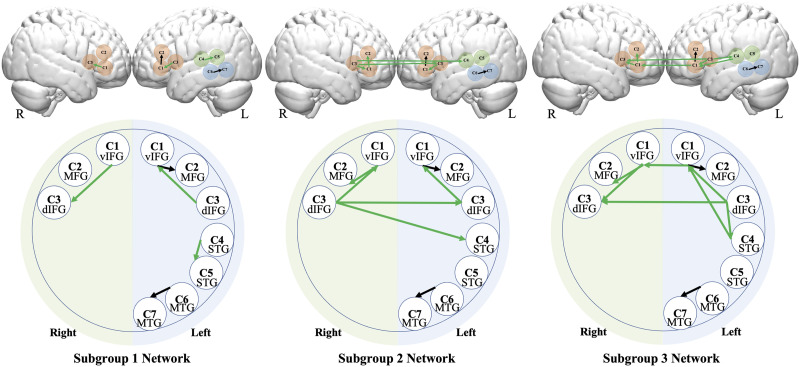
GIMME subgroup neural networks: The brain illustration (top row) and the map illustration (bottom row). Black lines indicate group-level connections; green lines indicate subgroup-level connections. Each circle represents a channel. IFG: inferior frontal gyrus; STG: superior temporal gyrus; MTG: middle temporal gyrus; d: dorsal; v: ventral.

GIMME identified two group-level connections that were shared by over 75% of participants. One was located between two left frontal channels: left C1 (IFG) and left C2 (MFG/IFG). The second group-level connection was located between the two left MTG channels (left C6 and C7; see Figure 3, black connections).

### Subgroup Neural Connectivity

**Figure 4.**
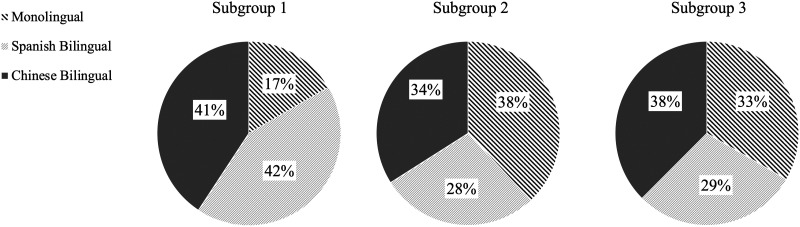
GIMME subgroup composition by language group.

Three subgroups emerged from the data driven GIMME search. Subgroups 1, 2, and 3 had approximately equivalent numbers of participants, *N* = 44, 51, and 47, respectively. Participants from the three language groups equally fell into the three subgroups, *χ*^2^(4) = 6.91, *p* = 0.141. Subgroup 1 had 9 monolinguals, 23 Spanish bilinguals, and 22 Chinese bilinguals. Subgroup 2 had 19 monolinguals, 14 Spanish bilinguals, and 17 Chinese bilinguals. Subgroup 3 had 16 monolinguals, 14 Spanish bilinguals, and 18 Chinese bilinguals (see Figure 4 for a pie chart display of the subgroup composition).

The subgroup-level connections are shown as green connections in Figure 4. Subgroup 1 had three subgroup-level connections: within left IFG (left C3–C1); within right IFG (right C3–C1); and between the two left STG channels (C4–C5). Subgroup 2 had five subgroup-level connections: within left IFG (left C1–C3); left and right contralateral IFG (bilateral C3); within right IFG (right C3–C1); right IFG and MFG (right C1–C2); and right IFG and contralateral left STG (right C3–left C4). Subgroup 3 had eight subgroup-level connections: left and right contralateral IFG (bilateral C1); left and right contralateral IFG (bilateral C3); within left IFG (left C3–C1); within right IFG (right C1–C3); right IFG and MFG (right C1–C2); left STG and IFG (left C5–C1); left IFG and STG (left C3–C5); the two left STG channels (left C4–C5).

In sum, for subgroup 1, the subgroup-level connections were exclusively within the same brain hub (i.e., within IFG or left STG); for subgroup 2, there were additional cross-lateral connections, especially among the phonological areas, such as between IFG and STG; and subgroup 3 had additional left-lateralized connections across brain hubs, such as between left IFG and STG. One-way ANOVA showed that the three subgroups differed significantly in their network density (i.e., number of connections), *F*(149, 2) = 138.6, *p* < 0.001, *η*^2^ = 0.65. Pairwise comparisons revealed that the three groups all differed from one another: subgroup 3 had the densest network compared to subgroup 2, followed by subgroup 1 (all *p*s < 0.001, Tukey-corrected).

To examine how GIMME subgroups may differ in the behavioral English tasks, we compared the English behavioral task proficiency among the three groups of participants controlled for age (see Table 3). Group 3 outperformed group 1 in the raw performances for all tasks except for phonological awareness (marginal insignificance, *p* = 0.050).

**Table 3.** Language and reading proficiency by GIMME subgroup

	Subgroup 1	Subgroup 2	Subgroup 3	ANOVA or Age-controlled ANCOVA	Pairwise comparison
	*M*(*SD*)	*M*(*SD*)	*M*(*SD*)	*p*	(Tukey-applied)
Age	7.35 (1.24)	7.66 (1.25)	8.04 (1.39)	0.032	G3 > G1*

fNIRS task accuracy (%)	76.43 (8.03)	79.00 (9.77)	81.91 (10.59)	0.005	G3 > G1**
Vocabulary	139.16 (27.98)	150.86 (28.85)	156.50 (30.52)	<0.001	G3 > G1***
					G2 > G1*
Phonological awareness	20.73 (7.29)	22.61 (7.23)	23.95 (7.82)	0.064	G3 > G1^+^
Morphological awareness	21.58 (10.94)	25.00 (10.46)	25.50 (10.96)	0.045	G3 > G1*
Single word reading	44.44 (15.91)	47.49 (15.78)	52.70 (14.32)	0.002	G3 > G1**
					G3 > G2*
Reading comprehension	23.56 (8.86)	25.96 (7.99)	27.84 (7.78)	0.003	G3 > G1**
Sentence reading fluency	30.68 (16.06)	36.63 (19.50)	42.83 (18.00)	<0.001	G3 > G1***
					G3 > G2*

*Note*. The analysis of covariance (ANCOVA) tests were age controlled except for the Age test, which used an ANOVA. ^+^*p* < 0.10, *p < 0.05, **p < 0.01, ***p < 0.001.

### Person-Specific Neural Network Density

#### Neural network density and English proficiency

Across all participants, children’s performance on all English measures, as estimated in raw scores, was significantly associated with children’s neural network density (*r*s = 0.21–0.32, *p*s < 0.011; Table 4). Notably, controlled for age, network density was still significantly associated with the score of reading fluency (*r* = 0.16, *p* = 0.045); while the associations with vocabulary, word reading, and reading comprehension did not reach significance (*r*s = 0.01–0.14, *p*s = 0.086–0.875; Table 4). Note that due to the highly correlated nature of the behavioral tasks, it may not be appropriate to apply a multiple comparison correction. However, if applied, the bivariate associations will generally survive multiple comparison corrections, while age-controlled associations may not.

**Table 4.** Correlation of neural network density with English language and reading proficiency

	Neural network density			
	Bivariate *r*	*p*	Age-controlled *r*	*p*
English vocabulary	0.25	0.004	0.08	0.322
English word reading	0.21	0.011	0.01	0.875
Passage comprehension	0.23	0.005	0.04	0.632
Sentence reading fluency	0.32	<0.001	0.16	0.045
Neuroimaging task accuracy	0.28	<0.001	0.14	0.086

#### Neural network density and heritage language proficiency

For Chinese bilingual children, Chinese word reading was significantly associated with children’s neural network density, controlling for age and English reading (B = 0.36, *p* = 0.026), whereas Chinese vocabulary was not a significant predictor of neural network density (B = 0.08, *p* = 0.577; Table 5). In contrast, as for Spanish bilingual children, Spanish vocabulary significantly predicted children’s neural network density controlling for age and English vocabulary (B = 0.43, *p* = 0.033), whereas Spanish reading was not a significant predictor (B = 0.28, *p* = 0.181; Table 5).

**Table 5.** Multiple regression predicting neural network density with Chinese/Spanish language and reading proficiency

	Neural network density							
	Chinese bilingual				Spanish bilingual			
	B	*t*	*p*	*R* ^2^	B	*t*	*p*	*R* ^2^
*Model 1 Vocabulary as the predictor*				0.161				0.125
Age	0.67	2.95	0.005		−0.10	−0.51	0.611	
English vocabulary	−0.35	−1.54	0.132		0.08	0.34	0.735	
Heritage language vocabulary	0.08	0.56	0.577		**0.43**	**2.20**	**0.033**	

*Model 2 Word reading as the predictor*				0.183				0.030
Age	0.33	1.54	0.131		0.09	0.44	0.664	
English word reading	−0.20	−0.96	0.340		−0.04	−0.17	0.866	
Heritage language word reading	**0.36**	**2.30**	**0.026**		0.28	1.36	0.181	

## DISCUSSION

Children’s unique language experiences lead to heterogeneous behavioral and neural profiles of language. Such individual variation makes it difficult to interpret group-level neuroimaging findings in child language, literacy, and bilingual development (Luk & Bialystok, 2013; Marian & Hayakawa, 2021). To advance our understanding of such heterogeneity, we used an innovative person-specific approach, GIMME, to identify variation in children’s neural networks for spoken word processing. The findings revealed that all participants, bilingual and monolingual children, formed short-distance neural connections within the left frontal and temporal regions, which are traditionally associated with word meaning retrieval and processing. Children who were older and more proficient in spoken and written English showed more long-distance connections within the broader language network and across the two hemispheres, suggesting that advancements in language skills are supported by more integrated neural networks (Hwang et al., 2013). Among bilinguals, those with stronger bilingual proficiency showed greater neural network density along the key regions of language processing, as a neurodevelopmental index of greater efficiency in cognitive processing (Schedlbauer et al., 2014). The findings inform theoretical perspectives aiming to link children’s cognitive and brain development by contextualizing the effects of heterogeneity in language experiences and proficiency on their emerging neural architecture for language and literacy.

### Shared Effects in the Neurobiology of Word Meaning Processes

Auditory word recognition builds upon the successful recognition of word sound and meaning constituents (Gwilliams, 2020; Perfetti & Hart, 2002). The present study employed a morpho-semantic word processing task that required children to dissect polysyllabic words into lexical morphemes and analyze the meanings of the morphemic units (i.e., bed*room*, class*room*, and mush*room*). We acknowledge that here and henceforth our discussion of the observed results refers to maximal anatomical overlays of the fNIRS channels (Hu et al., 2020). The findings revealed that >75% of all participants showed common short-distance connections linking left MTG subregions as well as IFG/MFG regions. MTG and IFG regions are commonly associated with semantic analysis and lexical retrieval (Binder, 2017; Fiorentino & Poeppel, 2007), whereas MFG is often associated with verbal working memory (Fegen et al., 2015; Gwilliams, 2020; Hagoort, 2019). Our findings thus support the idea that short-distance connections within left frontal and middle temporal regions play key roles in successful word processing by supporting morpho-semantic analyses that underlie spoken and written language development (Arredondo et al., 2015; Ip et al., 2017; Sun et al., 2023). These shared connections have implications for understanding the universality of language processing in children growing up in diverse linguistic contexts.

### Developmental Effects in Age and Proficiency Subgroups

GIMME identified three subgroups of participants with shared subgroup-level connections. Subgroup 1 exhibited the simplest network with three additional short-distance connections: one within right IFG, one within left IFG, and one within left STG regions. Subgroups 2 and 3 exhibited progressively more complex patterns with short- and long-distance connections. They were located between the right frontal and left temporal or between the left frontal and temporal regions. GIMME subgrouping was not related to children’s bilingual status, likely due to the fact that all participants in the current study were proficient English language users and attended English-only schools.

The subgrouping divisions correspond to children’s language and reading proficiency: Controlling for age, Subgroup 1 had the least advanced English language and reading ability and Subgroup 3 had the strongest competence. These findings suggest that language development is supported by both short- and long-distance connectivity in a child’s brain (Ouyang et al., 2017). Moreover, long-distance connections are likely critical in integrating different aspects of language processes such as phonological and morpho-semantic analyses (Li et al., 2014; Qi et al., 2019). Of special note, the left frontotemporal connection only existed in the most proficient Subgroup 3. This left vIFG–STG connection links regions of morpho-semantic and phonological analyses, likely reflecting the lexically abstract derivational morphemes in the current task (e.g., sing*er*, danc*er*, and fing*er*; Gwilliams, 2020; Sun et al., 2023). In sum, our findings suggest that children’s progress in word processing is supported by improvements in how the language network nodes integrate to support different elements of language subprocessing.

### Language Proficiency and Person-Specific Network Density

To understand how neural heterogeneity speaks to children’s behavioral profiles, we examined associations between neural network density and English language and reading proficiency across all participants. Prior work has linked low-density levels with early-life adversity and disease (e.g., Goetschius et al., 2020) whereas higher network density has been associated with greater efficiency in cognitive tasks (Arredondo et al., 2022; Schedlbauer et al., 2014). Therefore, we had expected that children with stronger language and reading competencies should exhibit greater network density along the key regions of language processing. This prediction was generally supported by the findings, especially when we looked at children’s raw score performance, including the in-scanner task accuracy (*r* = 0.30, *p* < 0.001) as well as the behavioral measures (*r*s = 0.21–0.32, *p*s ≤ 0.011). This brain–behavior association remained significant for sentence fluency controlling for age (although it should be noted that this may not survive multiple corrections due to the highly correlated nature among the behavioral tasks). This task requires a well-coordinated concert of word decoding, sentence comprehension, and cognitive monitoring skills, thus corresponding to a need for a more holistic neurocognitive network that the current channels have covered (Norton & Wolf, 2012). The findings for age-controlled scores for other tasks did not reach significance, likely due to the tightly interrelated nature of age and raw performance (Qi et al., 2021). Nevertheless, their validity is supported by both the sentence fluency task and the prior findings of positive associations between functional connectivity and language/reading proficiency (Finn et al., 2014; Qi et al., 2021; Skeide et al., 2016; Yu et al., 2018).

### Bilingual Proficiency and Person-Specific Network Density

To identify potential bilingual effects in children’s emerging neural networks for language, we examined the role of heritage language proficiency in their network density controlling for age and English proficiency. As heritage language measures differed across the two languages, the analyses were done for the Spanish- and Chinese-speaking groups separately. The analyses revealed significant contributions of heritage language proficiency to bilinguals’ neural network density, but in different aspects across the two bilingual groups. In Spanish bilinguals, the network density was associated with *Spanish vocabulary*, whereas in Chinese bilinguals, the network density was associated with *Chinese character reading*.

There are several possible explanations for these findings. Our English word processing functional task used in this study involves recognizing multimorphemic word units and the ability to dissect and comprehend words is critical for literacy success (Ehri, 1998; Goodwin et al., 2012); for bilingual learners, the properties of their home language may interact differently with English to influence this mechanism. The Spanish language contributes to English morpho-semantic skills through a cross-linguistic transfer at points of shared morphemic units including roots and affixes (Hernández et al., 2016). Prior behavioral data has shown that bilinguals with better Spanish vocabulary knowledge have better morphological literacy skills than English monolinguals and bilinguals who are less proficient in Spanish (Kuo et al., 2017). Our new neuroimaging findings suggest that children’s proficiency with Spanish vocabulary may facilitate their neural efficiency for processing morphologically complex English words, potentially via cross-linguistic transfer of shared morpho-semantic competencies.

In Chinese bilinguals, network density was positively associated with Chinese reading proficiency. Unlike Spanish-English bilingualism where speakers can enjoy the knowledge of cross-linguistically shared morphemic units, there are very few shared words between Chinese and English, as manifested by the null-to-small associations of vocabulary skills across the two languages (*r* = 0.10 according to a meta-analysis by Yang et al., 2017). Nevertheless, a critical element of Chinese literacy is that it is monosyllabic and Chinese characters reflect morphemes at the lexical level (McBride et al., 2022). Prior work has shown that Chinese-English bilinguals place greater reliance on morpho-semantic literacy skills and show enhanced neural activations of semantic processing during morpho-semantic tasks in English, relative to English monolinguals (Dong et al., 2020; Ip et al., 2017, 2019; Ruan et al., 2018; Sun et al., 2023; Sun, Zhang, Marks, Nickerson, et al., 2022). Structural neuroimaging research has found that Chinese-English bilinguals with better reading skills in both of their languages also had thicker left AF white matter tracts linking left IFG and STG regions (Gao et al., 2022). It is therefore possible that Chinese reading proficiency contributes to children’s neural efficiency for morphologically complex words in English, potentially via cross-linguistic transfer of morpho-syllabic literacy skills that are shared across bilinguals’ two languages.

### Theoretical Contributions and Inferences

Successful word recognition builds upon neurocognitive processes and integrations of word sound and meaning constituents. Therefore, neurodevelopmental frameworks pose that advancements in language faculty are supported by the emergence of networks that serve both specific and integrative language functions (Hickok & Poeppel, 2007; Werker & Hensch, 2015). Our findings advance these theoretical perspectives by demonstrating that school-age children have developed short-range neural connections that are specific to the word task at hand. More specifically, for our meaning-based task, most children demonstrated short-distance functional connectivity within the left MTG regions known for their key role in lexico-semantic processes, as well as within left IFG/MFG regions known to support analytical and cognitive demands for lexical tasks (Hagoort, 2019). Advancing beyond these short-distance connections, older and more proficient language learners built long-distance connections linking the critical regions of language functions, reflecting more integrated neural processes (Schedlbauer et al., 2014). In other words, our findings advance theories of language, cognition, and brain development by revealing the neurodevelopmental differences in language network quality and its association with literacy during elementary school years.

Language experiences differ across individuals. Bilingualism adds to the variability as children grow up with dual language experiences. Variations in bilingual experiences have long puzzled researchers who aim to identify core features of the elusive “bilingual brain” and its development (Claussenius-Kalman et al., 2021; Marian & Hayakawa, 2021). The present work leveraged this variability to better understand how individual differences contribute to bilingual language development and processing. Remarkably, the findings converged across two linguistically different bilingual groups: Spanish-English and Chinese-English bilingual children. Both groups showed greater network density in English in relation to their heritage language skills. The findings demonstrate that heritage language skills, even in languages as distinct as Spanish and Chinese, are related to children’s neural integration for language processing, a core characteristic of efficient language processes.

### Limitations

Several limitations should be considered when interpreting the current results. First, the sample included a wide age range, making it somewhat difficult to dissect the impacts of developmental maturity and skill proficiency. However, our analysis was able to parse out age, and the results, in general, revealed that both age and bilingual proficiency play significant roles in children’s neural network connectivity for English word processing. Future studies could recruit children at similar developmental stages to better obviate the effects of age. It is likely that for children of the same ages, those with higher language and reading proficiency also have a higher neural density within the broad language networks. Second, although the current study was able to recruit children with heterogeneous language experiences, the sample is still homogenous in many other aspects. For example, children were mostly from middle-class families and attended schools in southeast Michigan. Future studies could look to dissect neural network variation in groups that are diverse in these aspects such as socioeconomic backgrounds. Prior resting-state research has found that adolescents with childhood adverse experiences had sparser neural networks within the salience and default mode networks (Goetschius et al., 2020). It is therefore likely that lower-income socioeconomic backgrounds are associated with network sparsity within the brain regions for language. Third, the two bilingual groups were not fully equivalent in their heritage language reading proficiency, as the Spanish bilinguals on average had higher reading skills in Spanish than the Chinese bilinguals in Chinese. This is likely due to their English-dominant educational context, making it easier to transfer English literacy to Spanish than to Chinese. However, both groups were indeed competent in reading single words/characters in their heritage language, and their spoken language environments and proficiency were maximally equivalent.

### Conclusion

The study investigated sources of heterogeneity in children’s neural organization for spoken language skills that underlie both spoken and written language development. The findings revealed that, across participants, children’s English language proficiency was associated with their neural network characteristics, as manifested by the connectivity density within key brain regions of language processes. A more focal examination of the bilingual participants in the study further revealed that children’s dual-language proficiency was associated with their neural network characteristics, a finding that advances our understanding of the benefits of heritage language exposure and literacy instruction for children who speak a home language that is different from the society’s dominant languages. The findings thus highlight the importance of understanding not only group-level but also individual effects of language experience on the neural organization for cognitive function.

## ACKNOWLEDGMENTS

We are grateful to the families in Ann Arbor, Michigan, and the surrounding neighborhoods for their participation in our study. We also thank the research assistant team for their help with data collection.

## FUNDING INFORMATION

Ioulia Kovelman, National Institutes of Health (https://dx.doi.org/10.13039/100000002), Award ID: R01HD092498.

## AUTHOR CONTRIBUTIONS

**Xin Sun**: Conceptualization; Formal analysis; Investigation; Visualization; Writing – original draft; Writing – review & editing. **Rebecca A. Marks**: Conceptualization; Writing – review & editing. **Rachel L. Eggleston**: Investigation; Writing – review & editing. **Kehui Zhang**: Investigation; Writing – review & editing. **Chi-Lin Yu**: Investigation; Writing – review & editing. **Nia Nickerson**: Investigation; Writing – review & editing. **Valeria Caruso**: Investigation; Writing – review & editing. **Tai-Li Chou**: Writing – review & editing. **Xiao-Su Hu**: Investigation; Writing – review & editing. **Twila Tardif**: Writing – review & editing. **James R. Booth:** Writing – review & editing. **Adriene M. Beltz:** Methodology; Writing – review & editing. **Ioulia Kovelman:** Conceptualization; Funding acquisition; Methodology; Resources; Writing – review & editing.

## DATA AVAILABILITY STATEMENT

Data for the broader project are available on Deep Blue; see Sun, Zhang, Marks, Karas, et al. (2022, https://doi.org/10.1016/j.dib.2022.108048). For the current study, data and codes can be found at https://osf.io/uv3t6/?view_only=46569a15ebd241808a01d51f550c65dd.

## TECHNICAL TERMS

GIMME: Group iterative multiple model estimation; a network-mapping method that identifies connections of variables (i.e., brain signals) collected among multiple time points for each individual.
Heritage language: Language learned by its speakers at home as children, while they are often exposed to a different language outside home environments.

## References

[bib1] Arredondo, M. M., Ip, K. I., Shih Ju Hsu, L., Tardif, T., & Kovelman, I. (2015). Brain bases of morphological processing in young children. Human Brain Mapping, 36(8), 2890–2900. 10.1002/hbm.22815, 25930011 PMC5374976

[bib2] Arredondo, M. M., Kovelman, I., Satterfield, T., Hu, X., Stojanov, L., & Beltz, A. M. (2022). Person-specific connectivity mapping uncovers differences of bilingual language experience on brain bases of attention in children. Brain and Language, 227, Article 105084. 10.1016/j.bandl.2022.105084, 35176615 PMC9617512

[bib3] Beltz, A. M., & Gates, K. M. (2017). Network mapping with GIMME. Multivariate Behavioral Research, 52(6), 789–804. 10.1080/00273171.2017.1373014, 29161187 PMC6181449

[bib4] Beltz, A. M., & Molenaar, P. C. (2015). A posteriori model validation for the temporal order of directed functional connectivity maps. Frontiers in Neuroscience, 9, Article 304. 10.3389/fnins.2015.00304, 26379489 PMC4551081

[bib5] Beltz, A. M., Wright, A. G., Sprague, B. N., & Molenaar, P. C. (2016). Bridging the nomothetic and idiographic approaches to the analysis of clinical data. Assessment, 23(4), 447–458. 10.1177/1073191116648209, 27165092 PMC5104664

[bib6] Berken, J. A., Chai, X., Chen, J. K., Gracco, V. L., & Klein, D. (2016). Effects of early and late bilingualism on resting-state functional connectivity. Journal of Neuroscience, 36(4), 1165–1172. 10.1523/JNEUROSCI.1960-15.2016, 26818505 PMC6604829

[bib7] Bialystok, E., Craik, F. I., & Luk, G. (2012). Bilingualism: Consequences for mind and brain. Trends in Cognitive Sciences, 16(4), 240–250. 10.1016/j.tics.2012.03.001, 22464592 PMC3322418

[bib8] Binder, J. R. (2017). Current controversies on Wernicke’s area and its role in language. Current Neurology and Neuroscience Reports, 17, 58. 10.1007/s11910-017-0764-8, 28656532

[bib9] Brown, T. A. (2014). Confirmatory factor analysis for applied research. Guilford Press.

[bib10] Bulut, T. (2022). Neural correlates of morphological processing: An activation likelihood estimation meta-analysis. Cortex, 151, 49–69. 10.1016/j.cortex.2022.02.010, 35397379

[bib11] Cao, F., Bitan, T., & Booth, J. R. (2008). Effective brain connectivity in children with reading difficulties during phonological processing. Brain and Language, 107(2), 91–101. 10.1016/j.bandl.2007.12.009, 18226833 PMC2676797

[bib12] Chung, S. C., Chen, X., & Geva, E. (2019). Deconstructing and reconstructing cross-language transfer in bilingual reading development: An interactive framework. Journal of Neurolinguistics, 50, 149–161. 10.1016/j.jneuroling.2018.01.003

[bib13] Claussenius-Kalman, H., Hernandez, A. E., & Li, P. (2021). Expertise, ecosystem, and emergentism: Dynamic developmental bilingualism. Brain and Language, 222, Article 105013. 10.1016/j.bandl.2021.105013, 34520977

[bib14] Dong, Y., Tang, Y., Chow, B. W.-Y., Wang, W., & Dong, W.-Y. (2020). Contribution of vocabulary knowledge to reading comprehension among Chinese students: A meta-analysis. Frontiers in Psychology, 11, Article 525369. 10.3389/fpsyg.2020.525369, 33132948 PMC7561676

[bib15] Dotterer, H. L., Hyde, L. W., Shaw, D. S., Rodgers, E. L., Forbes, E. E., & Beltz, A. M. (2020). Connections that characterize callousness: Affective features of psychopathy are associated with personalized patterns of resting-state network connectivity. NeuroImage: Clinical, 28, Article 102402. 10.1016/j.nicl.2020.102402, 32891038 PMC7479442

[bib16] Dunn, D. M. (2019). Peabody Picture Vocabulary Test (5th ed.). Pearson.

[bib17] Dunn, L. M., & Dunn, L. M. (1998). The Peabody Picture Vocabulary Test—Revised (L. Lu & H. Liu, Trans.; Chinese ed.). Taipei: Psychology Publisher. (Original work published in 1997)

[bib18] Dunn, L., Padilla, E., Lugo, D., & Dunn, L. (1986). TVIP: Test Vocabolario Imágenes Peabody. Pearson.

[bib19] Ehri, L. C. (1998). Grapheme-phoneme knowledge is essential for learning to read words in English. In J. L. Metsala & L. C. Ehri (Eds.), Word recognition in beginning literacy (pp. 3–40). Routledge. 10.4324/9781410602718-6

[bib20] Enge, A., Friederici, A. D., & Skeide, M. A. (2020). A meta-analysis of fMRI studies of language comprehension in children. NeuroImage, 215, Article 116858. 10.1016/j.neuroimage.2020.116858, 32304886

[bib21] Fegen, D., Buchsbaum, B. R., & D’Esposito, M. (2015). The effect of rehearsal rate and memory load on verbal working memory. NeuroImage, 105, 120–131. 10.1016/j.neuroimage.2014.10.034, 25467303 PMC4267698

[bib22] Finn, E. S. (2021). Is it time to put rest to rest? Trends in Cognitive Sciences, 25(12), 1021–1032. 10.1016/j.tics.2021.09.005, 34625348 PMC8585722

[bib23] Finn, E. S., Shen, X., Holahan, J. M., Scheinost, D., Lacadie, C., Papademetris, X., Shaywitz, S. S., Shaywitz, B. A., & Constable, R. T. (2014). Disruption of functional networks in dyslexia: A whole-brain, data-driven analysis of connectivity. Biological Psychiatry, 76(5), 397–404. 10.1016/j.biopsych.2013.08.031, 24124929 PMC3984371

[bib24] Fiorentino, R., & Poeppel, D. (2007). Compound words and structure in the lexicon. Language and Cognitive Processes, 22(7), 953–1000. 10.1080/01690960701190215

[bib25] Francis, D., Carlo, M., August, D., Kenyon, D., Malabonga, V., Caglarcan, S., & Louguit, M. (2001). Test of Phonological Processing in Spanish. Center for Applied Linguistics.

[bib26] Friederici, A. D., Brauer, J., & Lohmann, G. (2011). Maturation of the language network: From inter- to intrahemispheric connectivities. PLOS ONE, 6(6), Article e20726. 10.1371/journal.pone.0020726, 21695183 PMC3113799

[bib27] Gagnon, L., Yücel, M. A., Dehaes, M., Cooper, R. J., Perdue, K. L., Selb, J., Huppert, T. J., Hoge, R. D., & Boas, D. A. (2012). Quantification of the cortical contribution to the NIRS signal over the motor cortex using concurrent NIRS-fMRI measurements. NeuroImage, 59(4), 3933–3940. 10.1016/j.neuroimage.2011.10.054, 22036999 PMC3279595

[bib28] Gao, Y., Meng, X., Bai, Z., Liu, X., Zhang, M., Li, H., Ding, G., Liu, L., & Booth, J. R. (2022). Left and right arcuate fasciculi are uniquely related to word reading skills in Chinese-English bilingual children. Neurobiology of Language, 3(1), 109–131. 10.1162/nol_a_00051 37215330 PMC10158580

[bib29] García-Pentón, L., Pérez Fernández, A., Iturria-Medina, Y., Gillon-Dowens, M., & Carreiras, M. (2014). Anatomical connectivity changes in the bilingual brain. NeuroImage, 84, 495–504. 10.1016/j.neuroimage.2013.08.064, 24018306

[bib30] Gates, K. M., & Molenaar, P. C. (2012). Group search algorithm recovers effective connectivity maps for individuals in homogeneous and heterogeneous samples. NeuroImage, 63(1), 310–319. 10.1016/j.neuroimage.2012.06.026, 22732562

[bib31] Goetschius, L. G., Hein, T. C., McLanahan, S. S., Brooks-Gunn, J., McLoyd, V. C., Dotterer, H. L., Lopez-Duran, N., Mitchell, C., Hyde, L. W., Monk, C. S., & Beltz, A. M. (2020). Association of childhood violence exposure with adolescent neural network density. JAMA Network Open, 3(9), Article e2017850. 10.1001/jamanetworkopen.2020.17850, 32965498 PMC7512058

[bib32] Goodwin, A., Lipsky, M., & Ahn, S. (2012). Word detectives: Using units of meaning to support literacy. The Reading Teacher, 65(7), 461–470. 10.1002/TRTR.01069

[bib33] Gwilliams, L. (2020). How the brain composes morphemes into meaning. Philosophical Transactions of the Royal Society B, 375(1791), Article 20190311. 10.1098/rstb.2019.0311, 31840591 PMC6939360

[bib34] Hagoort, P. (2019). The neurobiology of language beyond single-word processing. Science, 366(6461), 55–58. 10.1126/science.aax0289, 31604301

[bib35] Hernández, A. C., Montelongo, J. A., & Herter, R. J. (2016). Using Spanish-English cognates in children’s choices picture books to develop Latino English learners’ linguistic knowledge. The Reading Teacher, 70(2), 233–239. 10.1002/trtr.1511

[bib36] Hernandez, A. E., Claussenius-Kalman, H. L., Ronderos, J., Castilla-Earls, A. P., Sun, L., Weiss, S. D., & Young, D. R. (2019). Neuroemergentism: A framework for studying cognition and the brain. Journal of Neurolinguistics, 49, 214–223. 10.1016/j.jneuroling.2017.12.010, 30636843 PMC6326375

[bib37] Hickok, G. (2022). The dual stream model of speech and language processing. Handbook of Clinical Neurology, 185, 57–69. 10.1016/B978-0-12-823384-9.00003-7, 35078610

[bib38] Hickok, G., & Poeppel, D. (2007). The cortical organization of speech processing. Nature Reviews Neuroscience, 8(5), 393–402. 10.1038/nrn2113, 17431404

[bib39] Hu, X.-S., Wagley, N., Rioboo, A. T., DaSilva, A. F., & Kovelman, I. (2020). Photogrammetry-based stereoscopic optode registration method for functional near-infrared spectroscopy. Journal of Biomedical Optics, 25(9), Article 095001. 10.1117/1.JBO.25.9.095001, 32880124 PMC7463164

[bib40] Hwang, K., Hallquist, M. N., & Luna, B. (2013). The development of hub architecture in the human functional brain network. Cerebral Cortex, 23(10), 2380–2393. 10.1093/cercor/bhs227, 22875861 PMC3767958

[bib41] Ip, K. I., Hsu, L. S.-J., Arredondo, M. M., Tardif, T., & Kovelman, I. (2017). Brain bases of morphological processing in Chinese-English bilingual children. Developmental Science, 20(5), Article e12449. 10.1111/desc.12449, 27523024 PMC5309206

[bib42] Ip, K. I., Marks, R. A., Hsu, L. S.-J., Desai, N., Kuan, J. L., & Tardif, T. (2019). Morphological processing in Chinese engages left temporal regions. Brain and Language, 199, Article 104696. 10.1016/j.bandl.2019.104696, 31655417 PMC6876548

[bib43] Jasińska, K. K., Shuai, L., Lau, A. N. L., Frost, S., Landi, N., & Pugh, K. R. (2020). Functional connectivity in the developing language network in 4-year-old children predicts future reading ability. Developmental Science, 24(2), Article e13041. 10.1111/desc.13041, 33032375 PMC8186432

[bib44] Kovelman, I., Baker, S. A., & Petitto, L. A. (2008). Age of first bilingual language exposure as a new window into bilingual reading development. Bilingualism: Language and Cognition, 11(2), 203–223. 10.1017/S1366728908003386, 19823598 PMC2759761

[bib45] Kuo, L.-J., & Anderson, R. C. (2006). Morphological awareness and learning to read: A cross-language perspective. Educational Psychologist, 41(3), 161–180. 10.1207/s15326985ep4103_3

[bib46] Kuo, L.-J., Ramirez, G., de Marin, S., Kim, T.-J., & Unal-Gezer, M. (2017). Bilingualism and morphological awareness: A study with children from general education and Spanish-English dual language programs. Educational Psychology, 37(2), 94–111. 10.1080/01443410.2015.1049586

[bib47] Lane, S. [T.], Gates, K. [M.], Molenaar, P., Hallquist, M., & Pike, H. (2017). GIMME: Group iterative multiple model estimation [Computer software manual]. https://cran.r-project.org/web/packages/gimme

[bib48] Lane, S. T., Gates, K. M., Pike, H. K., Beltz, A. M., & Wright, A. G. C. (2019). Uncovering general, shared, and unique temporal patterns in ambulatory assessment data. Psychological Methods, 24(1), 54–69. 10.1037/met0000192, 30124300 PMC6433550

[bib49] Li, H., Xue, Z., Ellmore, T. M., Frye, R. E., & Wong, S. T. (2014). Network-based analysis reveals stronger local diffusion-based connectivity and different correlations with oral language skills in brains of children with high functioning autism spectrum disorders. Human Brain Mapping, 35(2), 396–413. 10.1002/hbm.22185, 23008187 PMC6869619

[bib50] Liu, H., & Cao, F. (2016). L1 and L2 processing in the bilingual brain: A meta-analysis of neuroimaging studies. Brain and Language, 159, 60–73. 10.1016/j.bandl.2016.05.013, 27295606

[bib51] Luk, G., & Bialystok, E. (2013). Bilingualism is not a categorical variable: Interaction between language proficiency and usage. Journal of Cognitive Psychology, 25(5), 605–621. 10.1080/20445911.2013.795574, 24073327 PMC3780436

[bib52] Marian, V., Bartolotti, J., Rochanavibhata, S., Bradley, K., & Hernandez, A. E. (2017). Bilingual cortical control of between-and within-language competition. Scientific Reports, 7(1), Article 11763. 10.1038/s41598-017-12116-w, 28924215 PMC5603581

[bib53] Marian, V., & Hayakawa, S. (2021). Measuring bilingualism: The quest for a “bilingualism quotient.” Applied Psycholinguistics, 42(S2), 527–548. 10.1017/S0142716420000533, 34054162 PMC8158058

[bib54] Marks, R. A., Labotka, D., Sun, X., Nickerson, N., Zhang, K., Eggleston, R. L., Yu, C.-L., Uchikoshi, Y., Hoeft, F., & Kovelman, I. (2022). Morphological awareness and its role in early word reading in English monolinguals, Spanish–English, and Chinese–English simultaneous bilinguals. Bilingualism: Language and Cognition, 26(2), 268–283. 10.1017/S1366728922000517 37063520 PMC10103835

[bib55] Marks, R. A., Sun, X., McAlister López, E., Nickerson, N., Hernandez, I., Caruso, V. C., Satterfiled, T., & Kovelman, I. (2022). Cross-linguistic differences in the associations between morphological awareness and reading in Spanish and English in young simultaneous bilinguals. International Journal of Bilingual Education and Bilingualism, 25(10), 3907–3923. 10.1080/13670050.2022.2090226 36714684 PMC9881678

[bib56] McBride, C., Pan, D. J., & Mohseni, F. (2022). Reading and writing words: A cross-linguistic perspective. Scientific Studies of Reading, 26(2), 125–138. 10.1080/10888438.2021.1920595

[bib57] Mohades, S. G., Struys, E., Van Schuerbeek, P., Mondt, K., Van De Craen, P., & Luypaert, R. (2012). DTI reveals structural differences in white matter tracts between bilingual and monolingual children. Brain Research, 1435, 72–80. 10.1016/j.brainres.2011.12.005, 22197702

[bib58] Muñoz-Sandoval, A. F., Woodcock, R. W., McGrew, K. S., & Mather, N. (2005). Batería III Woodcock-Muñoz. Riverside Publishing.

[bib59] Newman, E. H., Tardif, T., Huang, J., & Shu, H. (2011). Phonemes matter: The role of phoneme-level awareness in emergent Chinese readers. Journal of Experimental Child Psychology, 108(2), 242–259. 10.1016/j.jecp.2010.09.001, 20980019 PMC3644705

[bib60] NIRSOptix. 2018. CW6 system [Apparatus]. https://nirsoptix.com/CW6.html

[bib61] Norton, E. S., & Wolf, M. (2012). Rapid automatized naming (RAN) and reading fluency: Implications for understanding and treatment of reading disabilities. Annual Review of Psychology, 63(1), 427–452. 10.1146/annurev-psych-120710-100431, . 21838545

[bib62] Ouyang, M., Kang, H., Detre, J. A., Roberts, T. P., & Huang, H. (2017). Short-range connections in the developmental connectome during typical and atypical brain maturation. Neuroscience & Biobehavioral Reviews, 83, 109–122. 10.1016/j.neubiorev.2017.10.007, 29024679 PMC5730465

[bib63] Perfetti, C. A., & Hart, L. (2002). The lexical quality hypothesis. In L. Verhoeven, C. Elbro, & P. Reitsma (Eds.), Precursors of functional literacy (pp. 189–213). John Benjamins. 10.1075/swll.11.14per

[bib64] Price, R. B., Beltz, A. M., Woody, M. L., Cummings, L., Gilchrist, D., & Siegle, G. J. (2020). Neural connectivity subtypes predict discrete attentional-bias profiles among heterogeneous anxiety patients. Clinical Psychological Science, 8(3), 491–505. 10.1177/2167702620906149, 33758682 PMC7983837

[bib65] Qi, T., Schaadt, G., Cafiero, R., Brauer, J., Skeide, M. A., & Friederici, A. D. (2019). The emergence of long-range language network structural covariance and language abilities. NeuroImage, 191, 36–48. 10.1016/j.neuroimage.2019.02.014, 30738206

[bib66] Qi, T., Schaadt, G., & Friederici, A. D. (2021). Associated functional network development and language abilities in children. NeuroImage, 242, Article 118452. 10.1016/j.neuroimage.2021.118452, 34358655 PMC8463838

[bib67] Ruan, Y., Georgiou, G. K., Song, S., Li, Y., & Shu, H. (2018). Does writing system influence the associations between phonological awareness, morphological awareness, and reading? A meta-analysis. Journal of Educational Psychology, 110(2), 180–202. 10.1037/edu0000216

[bib68] Santosa, H., Zhai, X., Fishburn, F., & Huppert, T. (2018). The NIRS brain AnalyzIR toolbox. Algorithms, 11(5), 73. 10.3390/a11050073 PMC11218834 38957522

[bib69] Schedlbauer, A. M., Copara, M. S., Watrous, A. J., & Ekstrom, A. D. (2014). Multiple interacting brain areas underlie successful spatiotemporal memory retrieval in humans. Scientific Reports, 4(1), Article 6431. 10.1038/srep06431, 25234342 PMC4168271

[bib70] Schrank, F. A., McGrew, K. S., & Mather, N. (2014). Woodcock-Johnson IV Tests of Cognitive Abilities. Riverside.

[bib71] Skeide, M. A., Brauer, J., & Friederici, A. D. (2016). Brain functional and structural predictors of language performance. Cerebral Cortex, 26(5), 2127–2139. 10.1093/cercor/bhv042, 25770126

[bib72] Skeide, M. A., & Friederici, A. D. (2016). The ontogeny of the cortical language network. Nature Reviews Neuroscience, 17(5), 323–332. 10.1038/nrn.2016.23, 27040907

[bib73] Smith, S. M., Bandettini, P. A., Miller, K. L., Behrens, T. E. J., Friston, K. J., David, O., Liu, T., Woolrich, M. W., & Nichols, T. E. (2012). The danger of systematic bias in group-level FMRI-lag-based causality estimation. NeuroImage, 59(2), 1228–1229. 10.1016/j.neuroimage.2011.08.015, 21867760

[bib74] Smith, S. M., Miller, K. L., Salimi-Khorshidi, G., Webster, M., Beckmann, C. F., Nichols, T. E., Ramsey, J. D., & Woolrich, M. W. (2011). Network modelling methods for FMRI. NeuroImage, 54(2), 875–891. 10.1016/j.neuroimage.2010.08.063, 20817103

[bib75] Song, S., Su, M., Kang, C., Liu, H., Zhang, Y., McBride-Chang, C., Tardif, T., Li, H., Liang, W., Zhang, Z., & Shu, H. (2015). Tracing children’s vocabulary development from preschool through the school-age years: An 8-year longitudinal study. Developmental Science, 18(1), 119–131. 10.1111/desc.12190, 24962559 PMC4276547

[bib76] Su, M., Zhao, J., de Schotten, M. T., Zhou, W., Gong, G., Ramus, F., & Shu, H. (2018). Alterations in white matter pathways underlying phonological and morphological processing in Chinese developmental dyslexia. Developmental Cognitive Neuroscience, 31, 11–19. 10.1016/j.dcn.2018.04.002, 29727819 PMC6969203

[bib77] Sulpizio, S., Del Maschio, N., Fedeli, D., & Abutalebi, J. (2020). Bilingual language processing: A meta-analysis of functional neuroimaging studies. Neuroscience & Biobehavioral Reviews, 108, 834–853. 10.1016/j.neubiorev.2019.12.014, 31838193

[bib78] Sun, X., Li, L., Ding, G., Wang, R., & Li, P. (2019). Effects of language proficiency on cognitive control: Evidence from resting-state functional connectivity. Neuropsychologia, 129, 263–275. 10.1016/j.neuropsychologia.2019.03.020, 30951741

[bib79] Sun, X., Marks, R. A., Zhang, K., Yu, C.-L., Eggleston, R. L., Nickerson, N., Chou, T.-L., Hu, X.-S., Tardif, T., Satterfield, T., & Kovelman, I. (2023). Brain bases of English morphological processing: A comparison between Chinese-English, Spanish-English bilingual, and English monolingual children. Developmental Science, 26(1), Article e13251. 10.1111/desc.13251, 35188687 PMC9615011

[bib80] Sun, X., Zhang, K., Marks, R. [A.], Karas, Z., Eggleston, R., Nickerson, N., Yu, C.-L., Wagley, N., Hu, X., Caruso, V., Chou, T.-L., Satterfield, T., Tardif, T., & Kovelman, I. (2022). Morphological and phonological processing in English monolingual, Chinese–English bilingual, and Spanish–English bilingual children: An fNIRS neuroimaging dataset. Data in Brief, 42, Article 108048. 10.1016/j.dib.2022.108048, 35313503 PMC8933821

[bib81] Sun, X., Zhang, K., Marks, R. A., Nickerson, N., Eggleston, R. L., Yu, C.-L., Chou, T.-L., Tardif, T., & Kovelman, I. (2022). What’s in a word? Cross-linguistic influences on Spanish-English and Chinese-English bilingual children’s word reading development. Child Development, 93(1), 84–100. 10.1111/cdev.13666, 34570366 PMC8766884

[bib82] Thieba, C., Long, X., Dewey, D., & Lebel, C. (2019). Young children in different linguistic environments: A multimodal neuroimaging study of the inferior frontal gyrus. Brain and Cognition, 134, 71–79. 10.1016/j.bandc.2018.05.009, 30007529

[bib83] Wagley, N., Marks, R. A., Bedore, L. M., & Kovelman, I. (2022). Contributions of bilingual home environment and language proficiency on children’s Spanish–English reading outcomes. Child Development, 93(4), 881–899. 10.1111/cdev.13748, 35289947 PMC9619386

[bib84] Wagner, R. K., Torgesen, J. K., Rashotte, C. A., & Pearson, N. A. (1999). Comprehensive Test of Phonological Processing: CTOPP. Pro-ed.

[bib85] Wechsler, D. (2014). Wechsler Intelligence Scale for Children—Fifth Edition (WISC-V). The Psychological Corporation.

[bib86] Weiss-Croft, L. J., & Baldeweg, T. (2015). Maturation of language networks in children: A systematic review of 22 years of functional MRI. NeuroImage, 123, 269–281. 10.1016/j.neuroimage.2015.07.046, 26213350

[bib87] Werker, J. F., & Hensch, T. K. (2015). Critical periods in speech perception: New directions. Annual Review of Psychology, 66(1), 173–196. 10.1146/annurev-psych-010814-015104, 25251488

[bib88] Xiao, Y., Friederici, A. D., Margulies, D. S., & Brauer, J. (2016). Longitudinal changes in resting-state fMRI from age 5 to age 6 years covary with language development. NeuroImage, 128, 116–124. 10.1016/j.neuroimage.2015.12.008, 26690809 PMC4767215

[bib89] Yang, M., Cooc, N., & Sheng, L. (2017). An investigation of cross-linguistic transfer between Chinese and English: A meta-analysis. Asian-Pacific Journal of Second and Foreign Language Education, 2(1), Article 15. 10.1186/s40862-017-0036-9

[bib90] Yeatman, J. D., Dougherty, R. F., Rykhlevskaia, E., Sherbondy, A. J., Deutsch, G. K., Wandell, B. A., & Ben-Shachar, M. (2011). Anatomical properties of the arcuate fasciculus predict phonological and reading skills in children. Journal of Cognitive Neuroscience, 23(11), 3304–3317. 10.1162/jocn_a_00061, 21568636 PMC3214008

[bib91] Yu, X., Ferradal, S. L., Sliva, D. D., Dunstan, J., Carruthers, C., Sanfilippo, J., Zuk, J., Zöllei, L., Boyd, E., Gagoski, B., Ou, Y., Grant, P. E., & Gaab, N. (2021). Functional connectivity in infancy and toddlerhood predicts long-term language and preliteracy outcomes. Cerebral Cortex, Article bhab230. 10.1093/cercor/bhab230, 34347052 PMC10847903

[bib92] Yu, X., Raney, T., Perdue, M. V., Zuk, J., Ozernov-Palchik, O., Becker, B. L., Raschle, N. M., & Gaab, N. (2018). Emergence of the neural network underlying phonological processing from the prereading to the emergent reading stage: A longitudinal study. Human Brain Mapping, 39(5), 2047–2063. 10.1002/hbm.23985, 29380469 PMC5895515

