# Supplementary material for: Sources of Heterogeneity in Functional Connectivity During English Word Processing in Bilingual and Monolingual Children
Source: Neurobiol Lang (Camb). 2023 Apr 11;4(2):198–220. doi: 10.1162/nol_a_00092 (PMC10205148; doi:10.1162/nol_a_00092)

Supplementary Materials to manuscript

Sources of heterogeneity in functional connectivity in English word processing in bilingual and monolingual children

Table S1
English Lexical Decision Task Stimuli (Correct answers are bolded)

Block	Condition	Item	Target Word	Choice 1	Choice 2
1	Free roots/Compounds	1	airport	area	**airplane**
		2	human	**woman**	lemon
		3	motorcycle	recycle	**bicycle**
		4	car	**carseat**	carpet
2	Control	5	laundry	bookshelf	**laundry**
		6	napkin	giggle	**napkin**
		7	blanket	**blanket**	popcorn
		8	number	taxi	**number**
3	Affixes/Derivations	9	disagree	distance	**dishonest**
		10	running	ceiling	**jumping**
		11	skiing	**dancing**	morning
		12	warmer	**colder**	finger
4	Control	13	lady	**lady**	finish
		14	staple	frosting	**staple**
		15	textbook	**textbook**	maybe
		16	power	cartoon	**power**
5	Free roots/Compounds	17	caring	**careful**	carrot
		18	teammate	animate	**classmate**
		19	pencil	penguin	**penpal**
		20	camper	**camping**	camera
6	Affixes/Derivations	21	teacher	**doctor**	closer
		22	cutest	**coldest**	forest
		23	laughing	pudding	**joking**
		24	dirty	**muddy**	coffee
7	Affixes/Derivations	25	excitement	apartment	**amazement**
		26	heavy	**sleepy**	money
		27	reset	reading	**replay**
		28	walking	**pulling**	earring
8	Free roots/Compounds	29	winning	**winner**	window
		30	spaceship	**battleship**	friendship
		31	eyebrow	iphone	**eyelash**
		32	something	summer	**somewhere**
9	Control	33	explore	measure	**explore**
		34	question	**question**	after
		35	paddle	**paddle**	decide
		36	minute	children	**minute**
10	Free roots/Compounds	37	painter	**painting**	painful
		38	end	**weekend**	friend
		39	sunlight	polite	**flashlight**
		40	teacup	**teapot**	trex
11	Control	41	ground	story	**ground**
		42	country	**country**	dentist
		43	alarm	marker	**alarm**
		44	snowball	parrot	**snowball**
12	Affixes/Derivations	45	destroy	**detach**	dessert
		46	dancer	corner	**waiter**
		47	mistake	**misspell**	mister
		48	dislike	**disobey**	display

Figure S1
fNIRS cap configuration

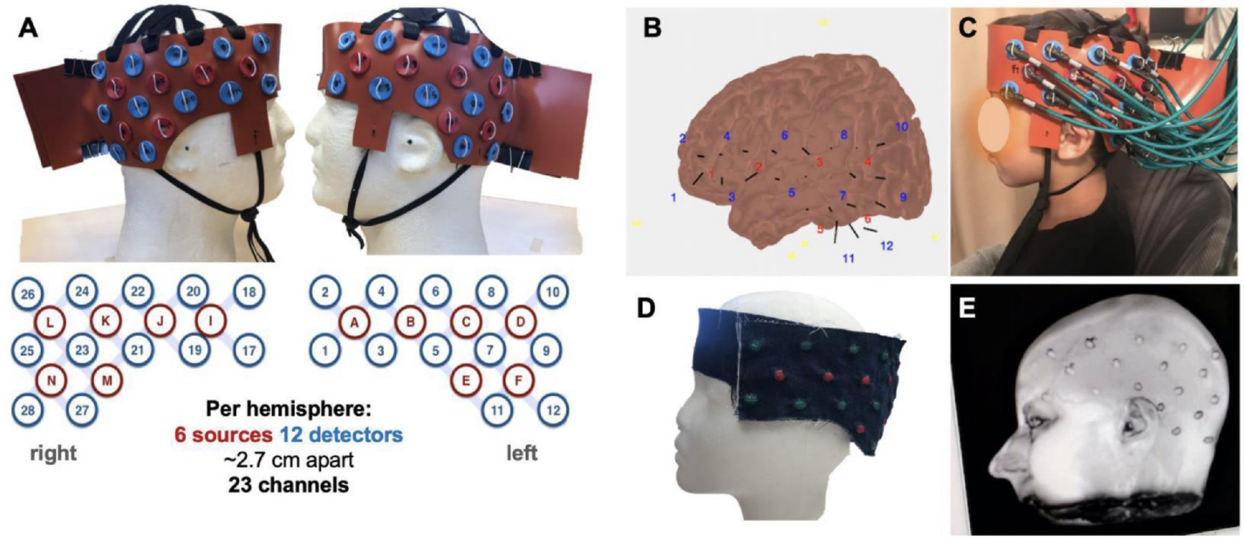

Note. (A) how signal (red, letters) and detector (blue, numbers) sensors are located on a silicone-rubber band around the participant's head, (B) surface map of the estimated brain regions covered by the cap design as digitized using AtlasViewer GUI (Aasted et al., 2015), (C) participant wearing the cap during data acquisition, (D) MRI version of the cap with vitamin-e capsules, and (E) visualization of vitamin-e capsules on the skull

Figure S2

Participants' Bilateral Brain Activation during Lexical Compound and Derivational Affixes Conditions (task > control) and direct comparisons of the two conditions (task > rest contrasts compared; all FDR adjusted $q < .05$)

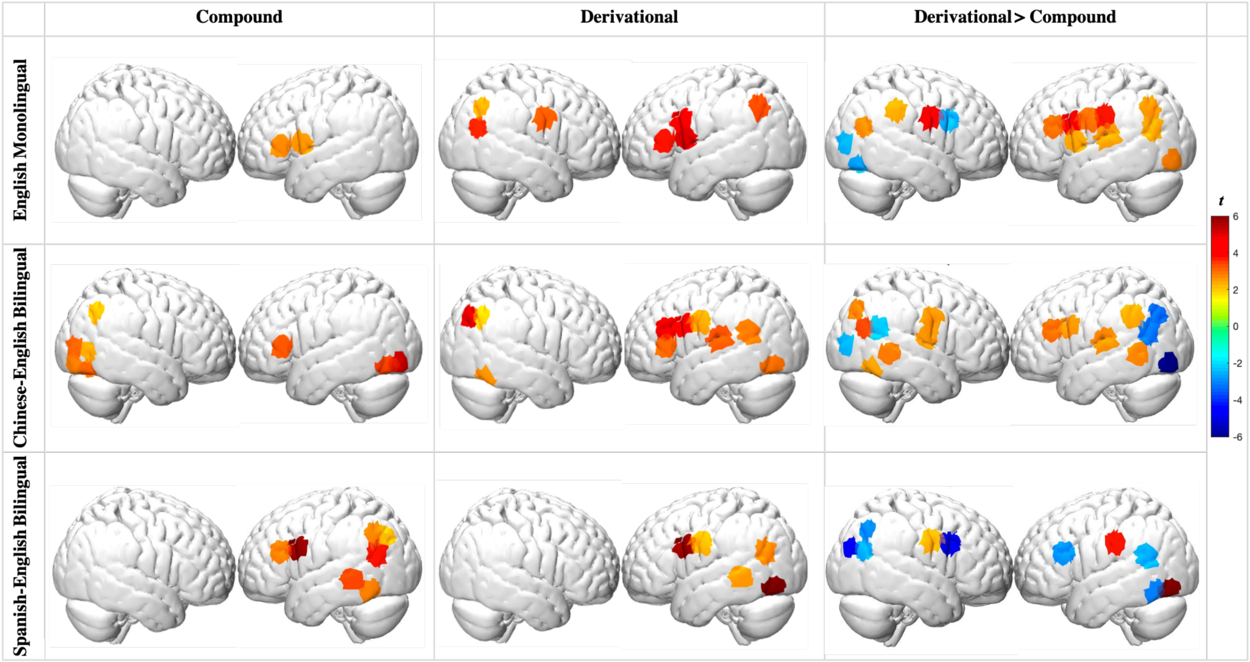

Supplement: Supplementary file 1 [file nol-4-2-198-s001.pdf]
